# Programmed cell death 4 as an endogenous suppressor of BDNF translation is involved in stress-induced depression

**DOI:** 10.1038/s41380-020-0692-x

**Published:** 2020-03-16

**Authors:** Yuan Li, Yufeng Jia, Dongdong Wang, Xiao Zhuang, Yan Li, Chun Guo, Hongxia Chu, Faliang Zhu, Jianing Wang, Xiaoyan Wang, Qun Wang, Wei Zhao, Yongyu Shi, Wanjun Chen, Lining Zhang

**Affiliations:** 1 Department of Immunology, School of Basic Medical Science, Shandong University, Jinan, China; 2 Research Institute of Neuromuscular and Neurodegenerative Diseases and Department of Neurology, Qilu hospital, Shandong University, Jinan, China; 3 Mucosal Immunology Section, National Institute of Dental and Craniofacial Research (NIDCR), US National Institutes of Health (NIH), Bethesda, MD USA

**Keywords:** Molecular biology, Neuroscience

## Abstract

Brain-derived neurotrophic factor (BDNF) is a growth factor that plays vital roles in the neuron survival, growth, and neuroplasticity. Alteration to BDNF expression is associated with major depressive disorder. However, the BDNF translational machinery in depression remains unknown. Herein, we pointed that Pdcd4, a suppressor oncogene, acted as an endogenous inhibitor for the translation of BDNF, and selectively repressed the translation of BDNF splice variant IIc mRNA in an eIF4A-dependent manner. Chronic restraint stress (CRS) up-regulated Pdcd4 expression in hippocampus via decreasing mTORC1-mediated proteasomes degradation pathway, which resulted in the reduction of BDNF protein expression. Moreover, over-expression of Pdcd4 in the hippocampus triggered spontaneous depression-like behaviors under the non-stressed conditions in mice, while systemic or neuron-specific knockout of Pdcd4 reverses CRS-induced depression-like behaviors. Importantly, administration of Pdcd4 siRNA or an interfering peptide that interrupts the Pdcd4-eIF4A complex substantially promoted BDNF expression and rescued the behavioral disorders which were caused by CRS. Overall, we have discovered a previously unrecognized role of Pdcd4 in controlling BDNF mRNA translation, and provided a new method that boosting BDNF expression through blocking the function of Pdcd4 in depression, indicating that Pdcd4 might be a new potential target for depressive disorder therapy.

## Introduction

Major depressive disorder (MDD) is a common and devastating illness, affecting ~7.4% of the populations in the world [1, 2]. It is also a lethal illness, for the increasing risk for suicide as well as cerebrovascular disorder and cardiac diseases [3]. Previous evidence points to an essential role of synaptic plasticity in MDD, in accord with the reduced synapse number in the prefrontal cortex (PFC) of the post-mortem subjects with depression, and researches in rodent models have confirmed that, like human depression, exposure to prolonged stress causes atrophy of neurons in the limbic brain regions [4–6]. Although disruption of complex neuroplasticity has been implicated in depression, the exact underlying mechanisms remain incompletely understood.

Brain-derived neurotrophic factor (BDNF) is an important factor relevant to neural plasticity, memory and emotional expression [7, 8]. Rodents were exposed to chronic stress, early life stress or unpredictable stress have significantly reduced BDNF expression in the hippocampus, resulting in impairment of synaptic plasticity and depression-like behaviors [9, 10]. Conversely, bilateral BDNF-infusions into the hippocampus led to antidepressant-like effects in preclinical animal models that could maintain for several days, suggesting that BDNF has an effect on antidepressant-like response [11]. Actually, the studies have shown that conventional antidepressant drugs, as well as selective serotonin reuptake inhibitors (SSRIs), enhanced BDNF mRNA expression and BDNF-TrkB receptor downstream signaling pathway [12–14]. Different with SSRI, ketamine, scopolamine and MK-801 (NMDA antagonist) have a rapid antidepressant effect in treatment-resistant patients. It has been found that a transiently increasing in hippocampal and cortical expression of BDNF protein paralleled the rapid antidepressant-like response of ketamine and scopolamine, inducing enhanced synaptic strength [15, 16]. Furthermore, the antidepressant effect of MK-801 attributes to ascending the translation of BDNF, which is mediated by the inactivation of eEF2 [17]. It indicates that not only the rapid antidepressant response is dependent on BDNF translation, but there is a possible translation relevant regulation of BDNF function in the development of depression, and rectify the dysfunction will contribute to the MDD treatment. Therefore, making clear of the BDNF translational machinery in depression is important for depression therapy.

Programmed cell death 4 (Pdcd4) is a novel tumor suppressor due to its role in inhibiting carcinogenesis, tumor progression and invasion [18, 19]. Pdcd4 interacts with translation initiation factor eIF4A and represses its RNA helicase activity. The co-crystal structures of the functional MA3 domains of Pdcd4 and eIF4A have revealed how Pdcd4 inhibited the translation initiation [20]. Pdcd4 inhibits translation in an mRNA-selective way, as the enzyme activity of eIF4A is thought to be necessary for unwinding secondary structure of 5’UTRs in certain oncogenic mRNAs. Pdcd4 also suppresses the translation of c-myb, but the inhibitory mechanism is not associated with eIF4A, which is determined by the RBD domain of Pdcd4 [21, 22]. Moreover, reports have also shown that Pdcd4 can be phosphorylated by S6K1, which is downstream of mTORC1, and thus participates in peptide elongation and dominates the neuronal complexity [23, 24]. However, the function of Pdcd4 in the CNS is rarely known.

Herein, we provided strong evidence that Pdcd4 deficiency conferred resilience to CRS-induced depression-like behaviors in mice via increasing mTORC1-regulated BDNF protein level and maintaining of synaptic plasticity in the hippocampus. While an interfering peptide that blocked the formation of Pdcd4-eIF4A complex substantially promoted BDNF expression and corrected the behavioral disorders which were caused by CRS. Together, our results reveal a distinct role of the tumor suppressor Pdcd4 in regulating neuronal plasticity in depressive disorders, and it might be a potential target for antidepressant treatment.

## Materials and methods

### Animals

The littermate wild type (WT) and Pdcd4-deficient male mice (all on C57BL/6 background) in this study have been previously described [25]. For behavioral test, *n* = 7 per No-CRS group, and *n* = 8 per CRS group. LoxP-flanked Pdcd4 mice were generated by Biocytogen Co., Ltd (Beijing, China) using CRISPR/Cas 9 and crossed with Eiia-Cre or CamkIIα-CreERT2 mice (Jax stock no. 012362) to yield systemic or neuron-specific Pdcd4 knockout mice. Different sgRNAs were designed for the target regions upstream of Pdcd4 exon 4, respectively. sgRNAs were constructed into the pCS (Puro) sgRNA/Cas9 expression plasmid (Biocytogen, Beijing, China) and the activity of sgRNAs were analyzed by the UCATM sgRNA/Cas9 plasmid construction and activity detection kit (Biocytogen, Beijing, China) according to the manufacturer’s protocol. The two sgRNAs with the best cutting activity were selected separately for the upstream and downstream target regions. In vitro transcripted sgRNAs and Cas9 mRNA were microinjected into the C57BL/6J single fertilized egg and produced the F0 mice. Chimeric mice were genotyped and crossed with C57BL/6 mice to obtain F1 mice carrying the heterozygous LoxP-flanked Pdcd4 gene. The genotype of mice was determined by DNA sequencing and PCR. The primers used for genotyping were as follows: forward: 5’- GTTTTGGTCTGCTGTGTTGGCAAGG-3’, reversed: 5’- ACTTCAACTCAACAAGTTGCTTGTCC -3’. To delete neuronal Pdcd4, 4–5-week-old mice received two doses of 10 mg tamoxifen (Sigma-Aldrich, MA, USA) in warm corn oil at two time points 48 h apart. Under non-stress condition, iCre group *n* = 7, ncKO group *n* = 8; under CRS condition, iCre group *n* = 14, ncKO group *n* = 14. Mice were housed 4–5 per cage and a circadian cycle of 12 h light and 12 h dark with an adequate food-water supply. All animal experiments and protocols were approved by the Animal Care and Utilization Committee of Shandong University.

### Chronic restraint stress

Male mice (6- to 8-week-old at the setup of experiments) were stressed daily during 9:00–11:00 a.m. for 14 days in the well-ventilated polypropylene restrainers without food and water. Then the behavioral testing was conducted 24 h after the last stressor to assess anxiety-like and depression behaviors.

### Behavioral testing

*Open field test*. Mice were placed in the area (40 × 40 × 35 cm, L × W × H) with 60 lux lighting and behavior was recorded for 10-min. A SMART video tracking system (Panlab, DC, USA) was used to analyze the traveled distance and the total time spent in the center area.

*Elevated plus maze*. The apparatus was black stainless steel with two closed and open arms (30 × 5 × 10 cm walls or 0.5 cm no wall) and set to a height of 50 cm beyond the ground. Mouse was placed into the center platform facing an open arm and a video tracking system (Panlab, DC, USA) was applied to score the time spent in open arms.

*Tail suspension test*. Each mouse was hung by the tail with tape (1 cm from tip) to a grid bar 30-cm height from the ground. Then the test was video-captured and last for 6 min. The time spent immobile (s) was counted; immobility was defined as the absence of escape-orientated movement.

*Force swimming test*. Mouse was individually placed in a glass cylinder (25 × 18 cm diameter) containing 15-cm water (22 ± 0.5 °C). The experiments were videotaped by a numeric tripod-fixed camera for tracking the behavior and the latency to immobility was scored. The time of immobility (s) was determined by the absence of movement except slight actions to maintain the head above the water.

*Sucrose preference test*. Mice were habituated to sucrose solution (1%) over 2 days. After deprivation of water for 22 h, mice were given free access to two bottles of water and 1% sucrose solution separately for 2 h. Then the fluid consumption was measured indicated by the weight loss of the bottles. The location of the two bottles was exchanged to avoid a side-bias during the next day test. Sucrose preference was calculated as follows: Sucrose preference (%) = sucrose intake/ total fluid consumption × 100%.

### Surgery and injection

Mice anesthetized with 5% chloral hydrate were implanted bilaterally with guide cannulas to the ventral hippocampus. The coordinates were as follows, vHIP: anteroposterior (AP), −2.54 mm; lateral (L), ±2.75 mm; dorsoventral (V), −2.0 mm. To prevent clogging, a stylus was placed into the cannula. 5 days with the mice recovery, the injection cannula was linked via PE20 tubing to a 10 µl Hamilton microsyringe motored by a microinjection pump (KDS 200, KD Scientific). Infusions were administered with a volume of 1 µl over the course of 2 min, and an additional 2 min was allowed for diffusion before the infusion cannulas were removed. TAT-NC or TAT-eIF4A_VI_ (1 mg/μl, 1 μl/lateral) was administered into the vHIP. For TST, FST experiments, TAT-NC injected mice were divided into two groups, No-CRS group *n* = 9, CRS group *n* = 10; TAT-eIF4A_VI_ injected group *n* = 11. For OFT, EPM experiments, TAT-NC injected mice were divided into two groups, No-CRS group *n* = 10, CRS group *n* = 8; TAT-eIF4A_VI_ injected group *n* = 9. For SPT, *n* = 18 per group.

*Virus microinjection*. Mice were anesthetized by 5% chloral hydrate (7.5 ml/kg, i.p.) and prepared for the injection of viruses liquid into the hippocampal by Hamilton micro-syringe with a microinjection pump (KDS 200, KD Scientific). Then the infection site was confirmed by the detection of GFP fluorescence via fluorescence microscopy (Olympus, Tokyo, Japan) and the expression of Pdcd4 was evaluated by western blot. The siRNA sequence for mouse Pdcd4 was as listed: antisense, 5′-GAGGCUAUGAGAGAAUUUATT-3′. Lentivirus of siPdcd4 and siNC were packaged and purified separately by Shanghai Genechem Co., Ltd (Shanghai, China). The AAV9 recombinant virus-containing OE-Pdcd4 or control was derived from ViGene Bioscience Company (Jinan, China). Control group *n* = 8, OE-Pdcd4 group *n* = 9.

*Drug administration*. Mice were randomly divided into four groups: Naïve + vehicle group, CRS + vehicle group, CRS + Rapamycin group and CRS + Pdcd4 KO + Rapamycin group, *n* = 7 per group. All agents were administered in a volume of 10 mg/kg, i.p. After 2 weeks of CRS exposure, animals were inoculated by a vehicle or Rapamycin (Med Chemexpress CO., Ltd, Monmouth Junction, USA) accordingly once every three days. K252a (dissolved in 1% DMSO, i.p. Sigma-Aldrich, MA, USA) was administrated once daily before CRS performance, *n* = 9.

*TAT-fused polypeptides*. Polypeptides containing 358rd to 365nd amino acid residues of eIF4A (HRIGRGGR-C, named TAT-eIF4A_VI_), which were fused to a TAT-like poly-arginine membrane permeability sequence (GRRRRRRRRRRR), were used in experiments. The peptides were synthesized and purified by GL Biochem (Shanghai, China).

### Plasmid construction

pEGFP-mBDNF encoding full-length BDNF (NM_007540.4) was obtained by subcloning the coding region of BDNF from mouse hippocampal cDNA into pEGFP-N1 (Invitrogen, Carlsbad, CA, USA). The BDNF expression vector containing BDNF UTRs and luciferase expression vectors pGL3 (Promega, Madison, MI, USA) containing I or IIc isoform BDNF UTRs were constructed with a template of mouse cDNA which was obtained by subcloning the coding region of BDNF. IIc 5’UTR deletion mutations (ΔLoop1: 1–67, ΔLoop2: 77–188, ΔLoop3: 217–282, ΔLoop4: 417–510) were generated using the KOD-Plus Mutagenesis Kit (Toyobo, Tokyo, Japan) according to the manufacturer’s protocol. The expression vector pcDNA3.1-mPdcd4 encodes full-length mouse Pdcd4 (NM_011050.4). The following expression vectors for mutant versions of mouse Pdcd4 were used: pcDNA3.1-Flag-mPdcd4-ΔRBD-1 and pcDNA3.1-Flag-mPdcd4-ΔRBD-2 encode RNA-binding deficient Pdcd4 proteins lacking amino acids 151–204 or 288–369, respectively. pcDNA3.1-Flag-mPdcd4-ΔMA3–1 and pcDNA3.1-Flag-mPdcd4-ΔMA3–2 encode eIF4A-binding deficient Pdcd4 proteins lacking amino acids 471–906 or 957–1347, respectively. The expression vector pcDNA3.1-heIF4A encodes full-length human eIF4A (NM_001416.3).

### Real-time PCR

Mice were sacrificed immediately after chronic resistant stress or baseline (No-CRS) Hippocampus (HIP) was removed after decapitation and PFC collection was acquired to use a mouse brain slicer (No-CRS, *n* = 8, CRS, *n* = 7). Total RNA was extracted by Trizol reagent (Tiangen, Beijing, China) following the manufacturer’s instructions. Then, the purified total RNA (500 ng) was reversely transcripted to cDNA using the RevertAid First Strand cDNA Synthesis Kit (Fermentas, Burlington, ON, Canada). Real-time PCR was performed in a Cycler (Bio-Rad, Hercules, CA, USA). The relative levels of mRNA were assessed through normalization by the β-actin mRNA levels. The primers were listed as follow:

Pdcd4 forward primer: 5’ –AAACAACTCCGTGATCTTTGTCCA- 3’and reverse primer: 5’ –TCAGGTTTAAGACGGCCTCCA- 3’;

BDNF forward primer: 5’ –TAAATGAAGTTTATACAGTACAGTGGTTCTACA- 3’and reverse primer: 5’ –AGTTGTGCGCAAATGACTGTTT- 3’;

β-actin forward primer: 5’ –CAACTTGATGTATGAAGGCTTTGGT- 3’and reverse primer: 5’ –ACTTTTATTGGTCTCAAGTCAGTGTACAG- 3’

### Immunofluorescence and Nissl staining

Mice were anesthetized by 5% chloral hydrate (7.5 ml/kg, i.p.), then, they were transcardially perfused with saline and 4% PFA. The brains were harvested, embedded in OCT, and sectioned 40 μm thick. The slides were washed by PBS for twice to remove OCT. For immunofluorescence, slides were incubated in 0.4% TritonX-100- diluted donkey serum to 10% which was used to block nonspecific staining for 30 min. Primary antibodies: anti-Pdcd4 (1:100), anti-NuN (1:500), anti-GFAP (1:500) were used to incubated with slides overnight at 4 °C, after that, they were washed with PBS three times and secondary antibody (1:500) incubated the slides at room temperature for 1 h. Washing in PBS three times, slides were mounted with cover glass, *n* = 4 per group. All of the images were captured with a Zeiss LSM780 confocal microscope (Oberkochen, Germany) at Microstructural Platform of Shandong University. Images were analyzed by NIH Image J. For Nissl staining, sections were cut at 40 μm intervals, and according to the standard procedure staining was done. Images were acquired with a light microscope Pannoramic 250 Flash III (3D Histech, Budapest, HUNGARY).

### Western blot and ELISA

Mice PFC and hippocampus were homogenized in lysis buffer with protease inhibitors and ready for western blot (*n* = 4 per group). Hippocampal lysate and cell-cultured medium were prepared for ELISA. The BDNF level was determined by BDNF ELISA Kit according to the instructions (Promega, Madison, MI, USA). The IL-10 and IL-6 ELISA protocols were referred on Kit instruction (Biolegend, San Diego, CA, USA). All the primary antibodies used were list in Supplementary Table 1.

### Luciferase assay

Luciferase activity was measured with a dual luciferase assay system (Promega, Madison, WI, USA) in HEK293 cells with siRNA or overexpression plasmid, and the readout was determined using a microplate luminometer (Centro LB 960; Berthold, Wildbad, Germany). Three independent experiments are shown.

### RIP assay

Mice hippocampus tissue was lysed in RNP-IP buffer (150 mM NaCl, 50 mM Tris-Cl pH7.5, 1% NP-40, 0.5% Sodium deoxycholate, 0.05% SDS, 1 mM EDTA(pH8.0), 1× Protease Inhibitor Cocktail (Sigma-Aldrich, MA, USA), 40 U/ml RiboLock RNase Inhibitor (Thermo, CA,USA) in DEPC water. The material was used for immunoprecipitation for 8 h at 4 °C with rabbit anti-Pdcd4 antibody, anti-SNRNP70 antibody or rabbit IgG. Following that the samples were washed five times with washing buffer (150 mM NaCl, 50 mM Hepes pH 7.5, 6 mM EGTA, 1 mM EDTA, 0.5% NP-40, protease inhibitor mixture and RNase inhibitor). After elution the beads, the samples were recovered cross-linking at 65 °C for 4 h. Further added with proteinase K administration for 1 h at 37 °C, followed by RNase-free DNase I for 15 min at room temperature. RNA was purified by TRIzol extraction. cDNA synthesis and RT-PCR were performed as described. All the experiments were repeated for three times.

### Co-immunoprecipitation

HEK293 cells were transfected with plasmids and peptides in lipo2000. Cell protein were extracted 24 h later with TNE buffer (10 mM Tris, 150 mM NaCl, 1 mM EDTA, 1% NP-40, 10% glycerol and protease inhibitors). The cell lysates were precipitated with M2-Sepharose (Sigma-Aldrich, MA, USA) or Protein A/G beads (Santa Cruz, Dallas, Texas, USA) overnight at 4 °C. The beads were rinsed five times with the TNE buffer and boiled in sample buffer (Invitrogen, Carlsbad, CA, USA) for SDS-PAGE.

### Bimolecular fluorescence complementation, BiFC assay

mPdcd4 and heIF4A full-length sequences were constructed into pBiFC-VC155 vector and pBiFC-VN155 vector, individually. The plasmids were transfected into HEK293 cells with TAT-NC or TAT-eIF4A_VI_ peptides. Cells cultured for 24 h and were fixed with 4% paraformaldehyde. Then, the samples were washed three times with PBS and mounted on slides in ProLong Gold medium (Invitrogen, Carlsbad, CA, USA). All of the images were captured with a Zeiss LSM780 confocal microscope fitted with a 63× oil-immersion objective lens (Microstructural Platform of Shandong University). Three independent experiments are shown.

### Golgi staining

Mice were anesthetized by 5% chloral hydrate (7.5 ml/kg, i.p) and the fresh brains were scratched from skull ready for the manufacture instruction of TM FD Rapid GolgiStain Kit (FD NeuroTechnologies, MD, USA). Brain slice was cut at room temperature (150 μm sections) on a vibratome (VT1200S, Leica, Germany). Slides were immersed in DDW three times for 5 min after air dry for 48 h and then transferred into a solution of D and E (Golgi kit) for 5–10 min at 4 °C followed by rinse three times for 5 min each in DDW. They were then dehydrated with ethanol, cleared with Histoclear (three times for 5 min each), and cover-slipped with DPX mounting medium, *n* = 4 per group. Z-stack pictures (20×) were captured by Pannoramic 250 Flash III (3D Histech, Budapest, Hungary).

### GFP positive neurons dendritic spine analysis

Dendritic segments of 100 μm in length were scanned by laser-scanning confocal microscopy (Zeiss LSM780 confocal microscope, Microstructural Platform of Shandong University). GFP-positive DG layer neurons were identified by blinding to experimental conditions. ZEN configuration was applied to raw three-dimensional digital images, which were then analyzed for spine density. Individual spines were measured manually for head diameter, length and neck diameter from image z-stacks. Measurement was blinded to all experimental conditions, *n* = 4 per group.

### Cell culture and transfection

The human embryonic kidney (HEK-293T) cells were brought from Shanghai Cell Bank of Chinese Academy of Sciences (Shanghai, China). Cells were grown in DMEM supplemented with 10% fetal bovine serum (FBS) at 37 °C in a humidified atmosphere of 5% CO2 in air. Cells were transfected with lipofectamine 2000 (Invitrogen, Carlsbad, CA, USA). After 24 h, the cells were lysed by RIPA buffer.

*Primary hippocampal neurons culture*. Dissociated cultures of mice hippocampal were prepared from embryonic day 17.5 (E17.5) C57/BL mice. Briefly, hippocampi were extracted from the embryos and incubated for 20 min in 0.25% trypsin-EDTA. Digested tissues were plated on plates which were coated with 0.1 mg/ml poly-D-lysine (Sigma-Aldrich, MA, USA). Neurons-cultured medium consisted of Neurobasal medium (Invitrogen, Carlsbad, CA, USA) supplemented with 2% B27 and GlutaMax (Invitrogen, Carlsbad, CA, USA). An incubator with 5% CO_2_, and temperature at 37 °C was utilized for neuron culture.

### Microarray data analysis

Brains from tetrads of subjects with schizophrenia, major depression, or bipolar disorder and unaffected comparison subjects data were obtained for GEO (GSE53987, GSE42546 and GSE12654), and data were normalized by Robust Multi-array Average (RMA) [26–28]. Fold change of the data were normalized to Control. The detail information of Postmortem human brain tissue including brain region, and disease group age, sex, postmortem interval (PMI), manner of death were illustrated in Supplementary Tables 2 and 3.

### Statistical analysis

Data were displayed as the mean ± SEM and analyzed by using Graphpad Prism 7.0 (GraphPad Software, San Diego, CA, USA). In all behavioral experiments, the animals were randomly divided into different group which was based on the table of random digit, and observers were blinded to the genotypes and treatments during the experiments. Normal distributions were compared by using the Kolmogorov–Smirnov test. All data were subjected to standard normality tests, and variance between groups was assessed. Two-way ANOVA (No-CRS vs. CRS × WT vs. KO or No-CRS vs. CRS × siNC vs. siPdcd4) followed by Sidak’s multiple comparison test were performed for CRS-induced depression-like behavior and all measures of biochemistry from tissues. One-way ANOVA followed by Tukey’s post hoc test were applied for analyzing conditional knockout mice behavior under CRS condition and peptide-injected mice behavior analysis. Unpaired Student’s *t* tests were performed for Pdcd4- overexpressed mice depression-like behavior. Statistical tests were two-sided and *p < 0.05, **p < 0.01 was regarded statistically significant. All data points provided are biological replicates and represent *n*. Exclusion data were defined when >2 standard deviations from the mean. Sample size estimates were conducted based on previous studies about the effects of CRS on behavior [29], and were relied on an expected effect size of 0.8 standardized unit with 80% power and 5% significance level.

## Results

### Deletion of Pdcd4 prevents CRS-induced depression-like behaviors in mice

**Fig. 1:**
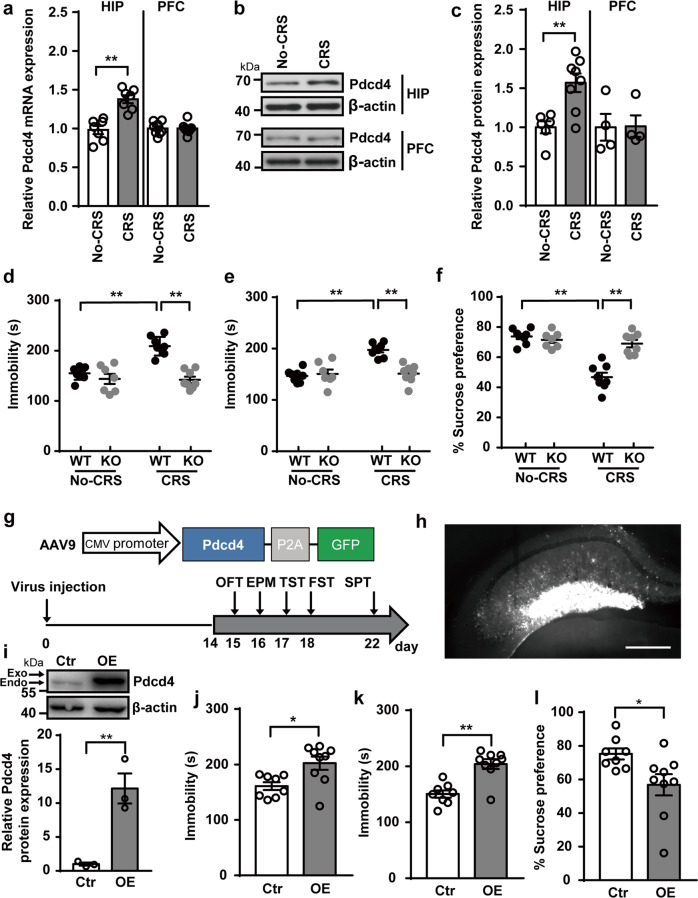
Deletion of Pdcd4 prevents CRS-induced depression-like behaviors in mice. **a** The change of mRNA levels of Pdcd4 in the hippocampal (HIP) and prefrontal cortex (PFC) after CRS. No-CRS *n* = 7–8, CRS *n* = 7–8; unpaired two-tailed Student’s *t* test, **P < 0.01. **b**, **c** The change of protein levels of Pdcd4 in the HIP and PFC after CRS. For HIP No-CRS *n* = 6, CRS *n* = 8, for PFC No-CRS *n* = 4, CRS *n* = 4; unpaired two-tailed Student’s *t* test, **P < 0.01. **d** Immobility time in TST, **e** immobility time in FST, and **f** sucrose consumption in SPT under basal or stress condition. *n* = 8 per group; mean ± SEM, two-way ANOVA and Sidak’s multiple comparison test, **P < 0.01. **g** Top, schematic of construct displaying mouse Pdcd4 subcloned into an AAV9 plasmid under transcriptional regulation of the CMV promoter (AAV9-CMV-mPdcd4-P2A-GFP); AAV9-CMV-P2A-GFP served as the control. Bottom, the schematic of the experiment. **h** Representative photomicrographs of injection sites in the hippocampus. *n* = 4 per group, Scale bars, 100 µm. **i** Fold change of Pdcd4 protein from hippocampus microdissections from control (*n* = 4) or AAV-Pdcd4–injected (*n* = 3) mice, unpaired two-tailed Student’s *t* test. **j** Mean immobility time (±SEM) for control (*n* = 8) and OE-Pdcd4 (*n* = 9) animals in the tail suspension test, unpaired two-tailed Student’s *t* test. **k** Mean immobility time (±SEM) for control (*n* = 8) and OE-Pdcd4 (*n* = 9) animals in the force swimming test, unpaired two-tailed Student’s *t* test. **l** Mean sucrose preference (±SEM) for control (*n* = 8) and OE-Pdcd4 (*n* = 9) animals, unpaired two-tailed Student’s *t* test. *P < 0.05 and **P < 0.01.

To study the role of Pdcd4 in the central nervous system (CNS), we first determined the expression of Pdcd4 in the brain in mice. Pdcd4 was widely distributed in the brain, including the PFC, hippocampus, hypothalamus, striatum, entorhinal cortex and the thalamus (Supplementary Fig. 1a). Pdcd4 was mainly expressed by the NeuN positive neurons, but not the GFAP positive astrocytes as shown in the PFC and hippocampal CA1 regions (Supplementary Fig. 1b, c). In CRS-treated wild-type (WT) mice, both the levels of Pdcd4 mRNA and protein were significantly up-regulated in the hippocampus, but intriguingly not in the PFC (Fig. 1a–c). In the hippocampus, Pdcd4 expression was mostly increased within the CA1, CA3 pyramidal cell layers and the DG granular cell layer after CRS (Supplementary Fig. 1d, e). To investigate whether Pdcd4 was involved in CRS-induced depression-like behaviors, we applied the Pdcd4 knockout (KO) mice into the CRS paradigm. The gross morphology of the hippocampus was unaffected by the loss of Pdcd4 (Supplementary Fig. 2a–e). Under non-stressed conditions, Pdcd4 KO mice did not display any difference in anxiolytic and depressive-like behaviors compared with WT mice (Fig. 1d–f, Supplementary Fig. 3a–c). As expected, after CRS, littermate WT mice showed significantly increased depression-like behaviors characterized by increased immobility in the tail suspension test (TST) and force swimming test (FST), and decreased sucrose consumption in the sucrose preference test (SPT). Strikingly, the response to CRS was completely absent in Pdcd4 KO mice (Fig. 1d–f). Further, we also investigated the effect of Pdcd4 KO on CRS-induced anxiety-like behaviors. WT mice displayed obviously increased anxiety-like behavior after CRS as shown by decreased time spent in the center of the open field test (OFT) and in the open arm of the elevated plus maze (EPM). However, the CRS effects were also absent in Pdcd4 KO mice in response to CRS (Supplementary Fig. 3a–c). Altogether, these data suggest that Pdcd4 deficiency in mice show resistance to CRS-induced depression- and anxiety-like behaviors.

### Overexpression of Pdcd4 in the hippocampus increases depression- and anxiety-like behaviors

To further validate the causal relationship between the expression of Pdcd4 and depressive disorder, a recombinant AAV9 virus carrying mouse Pdcd4 gene and GFP (OE-Pdcd4) was bilaterally injected into the hippocampus of mice, the control group received an AAV9 virus only expressing GFP (Fig. 1g). The diffusion of virus was detected by immunofluorescent staining of GFP at day 14 after injection and was observed to be widely distributed in the ventral hippocampus, especially in the dentate gyrus (DG) (Fig. 1h). Western blotting analysis also confirmed the overexpression of Pdcd4 in the hippocampus (Fig. 1i). Next, we investigated whether the enhanced Pdcd4 expression could mimic the increased depression- and anxiety-like behaviors caused by CRS under physiological conditions. Mice with Pdcd4 overexpression showed significantly increased immobility in FST and TST and decreased sucrose consumption in SPT compared with the control group (Fig. 1j–l). Furthermore, OE-Pdcd4-treated mice spent less time in the central area of the open field and in the open arms in EPM compared with mice in the control group (Supplementary Fig. 3d–f). Thus, these results indicate that Pdcd4 overexpression in the hippocampus triggers spontaneous depression- and anxiety-like behaviors even under the non-stressed conditions.

### Pdcd4 mediates CRS-induced synaptic plasticity impairment in hippocampus through blocking mTORC1-regulated BDNF signaling

**Fig. 2:**
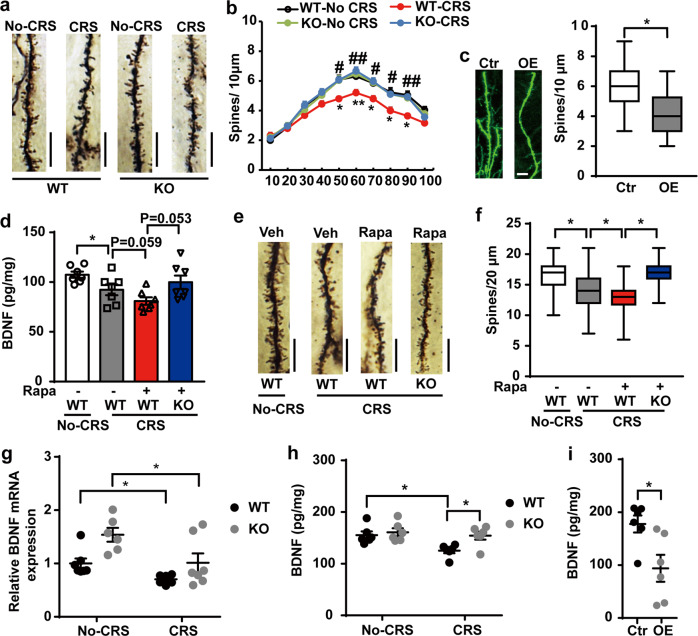
Pdcd4 mediates CRS-induced synaptic plasticity impairment in hippocampus through blocking mTORC1-regulated BDNF signaling. **a** Representative photomicrographs of dendritic spines from DG granular cells, scale bar, 10 μm. **b** Spine density in dendrites of DG granular cells in Pdcd4 KO and WT mice. *n* = 4 per group; mean ± SEM, two-way ANOVA and Sidak’s multiple comparison test, *P < 0.05, **P < 0.01 vs. the No-CRS group; ^#^*P* < 0.05, ^##^*P* < 0.01 vs. the CRS-WT group. **c** Representative images of high-magnification z-stack projections of segments of the DG dendrites (scale bar, 5 µm). Mean ± SEM of spine density from control (*n* = 36 from four mice), OE-Pdcd4 (*n* = 46 from four mice). *P < 0.05 relative to control mice (unpaired two-tailed Student’s *t* test). **d** Mice hippocampus BDNF was detected by ELISA. *n* = 6–7 per group, Rapamycin (Rapa), mean ± SEM, unpaired two-tailed Student’s *t* test, *P < 0.05. **e** Representative photomicrographs of dendritic spines from DG granular cells, scale bar, 10 μm. **f** Spine density in dendrites of DG granular cells. *n* = 4 per group; mean ± SEM, unpaired two-tailed Student’s *t* test, *P < 0.05. **g** Quantitative RT-PCR analysis of BDNF mRNA expression in the hippocampus in Pdcd4 KO and WT mice. *n* = 6–7 per group; mean ± SEM, two-way ANOVA (WT vs. KO, F_1,22_ = 10.97, *P* < 0.01; No-CRS vs. CRS, F_1,22_ = 11.67, *P* < 0.01; interaction, F_1,22_ = 0.846, *P* = 0.3676) and Sidak’s multiple comparison test, *P < 0.05. **h** Quantitative ELISA analysis of pan-BDNF protein expression in the hippocampus in Pdcd4 KO and WT mice. *n* = 6 per group; mean ± SEM, two-way ANOVA (WT vs. KO F_1,20_ = 6.141, *P* < 0.05; No-CRS vs. CRS, F_1,20_ = 6.906, *P* < 0.05; interaction, F_1,20_ = 2.816, *P* = 0.1089) and Sidak’s multiple comparison test, *P < 0.05. **i** Quantitative ELISA analysis of pan-BDNF protein expression in the hippocampus in OE-Pdcd4 and Control mice. *n* = 6 per group; mean ± SEM, unpaired two-tailed Student’s *t* test, *P < 0.05 vs. the control group.

We next investigated whether Pdcd4 participated in depression via regulating the spine formation of hippocampal neurons by Golgi staining. There was no significant difference in spine density between Pdcd4 KO and WT mice under the baseline. However, the number of spines in DG granule neurons was markedly reduced in WT mice but not in the Pdcd4 KO mice after CRS, suggesting that deletion of Pdcd4 reversed the CRS-induced spine loss (Fig. 2a, b). Conversely, overexpression of Pdcd4 led to significant reduction of spine numbers in granule neurons of DG (Fig. 2c). These data indicate that Pdcd4 mediates CRS-induced synaptic plasticity impairment in the hippocampus.

Since the activation of the mTORC1 signaling promoted phosphorylation and ubiquitin-mediated degradation of Pdcd4 [24, 30], we thus speculated that CRS might up-regulate the level of Pdcd4 by inhibiting the mTORC1 signaling-mediated degradation of Pdcd4. The result showed that CRS significantly reduced the activation of mTORC1 and decreased the phosphorylation of Pdcd4 (S67) (Supplementary Fig. 4a). As increased phosphorylation of Pdcd4 is associated with its ubiquitin-mediated degradation, we next confirmed the ubiquitination of Pdcd4 in the hippocampus. CRS significantly decreased the ubiquitination of Pdcd4 in the hippocampus, indicating reduced ubiquitin-dependent degradation of Pdcd4 (Supplementary Fig. 4b). Notably, the alteration of Pdcd4 was a long-term effect of chronic stress, as acute restraint stress (ARS) did not change the phosphorylation of mTORC1 and Pdcd4 expression (Supplementary Fig. 4c, d). These results indicate that CRS inhibits ubiquitin-mediated degradation of Pdcd4 through attenuating the activation of mTORC1 and phosphorylation of Pdcd4.

BDNF is a crucial neurotropic factor mediating neural plasticity and the mTORC1 signaling has been shown to contribute to BDNF synthesis [17, 31]. Since Pdcd4 has been considered as a translational repressor, we hypothesized that Pdcd4 might work as an intermediate molecule in mTORC1-regulated BDNF expression in response to CRS. To confirm this, we injected the mTORC1 inhibitor, rapamycin, into WT and Pdcd4 KO mice, respectively, and examined the levels of mTORC1 phosphorylation and BDNF expression. CRS resulted in the decreased phosphorylation of mTORC1 and of its downstream effective molecule, S6, and then increased Pdcd4 expression in the hippocampus compared with the non-stressed WT mice, which could be further enhanced by rapamycin (Supplementary Fig. 5a). Strikingly, Pdcd4 deficiency abolished CRS and rapamycin-induced reduction of BDNF expression (Fig. 2d). The deletion of Pdcd4 prevented both CRS- and rapamycin-induced spine loss of DG granule neurons in the hippocampus, in accordance with the alteration of BDNF (Fig. 2e, f). These data suggest a vital role of Pdcd4 in mediating mTORC1-regulated BDNF expression.

To explore the regulatory role of Pdcd4 on BDNF, we examined the BDNF mRNA and protein levels after CRS. CRS significantly reduced the level of BDNF mRNA both in the WT and Pdcd4 KO mice (Fig. 2g). However, the level of BDNF protein was only down-regulated in WT mice after CRS but not in the Pdcd4 KO mice after CRS (Fig. 2h). Conversely, overexpression of Pdcd4 in the hippocampus also led to lower BDNF protein expression (Fig. 2i). These results suggest that Pdcd4 regulates BDNF expression at the post-transcriptional level.

### Pdcd4 selectively represses the translation of BDNF splice variant IIc mRNA in an eIF4A-dependent manner

**Fig. 3:**
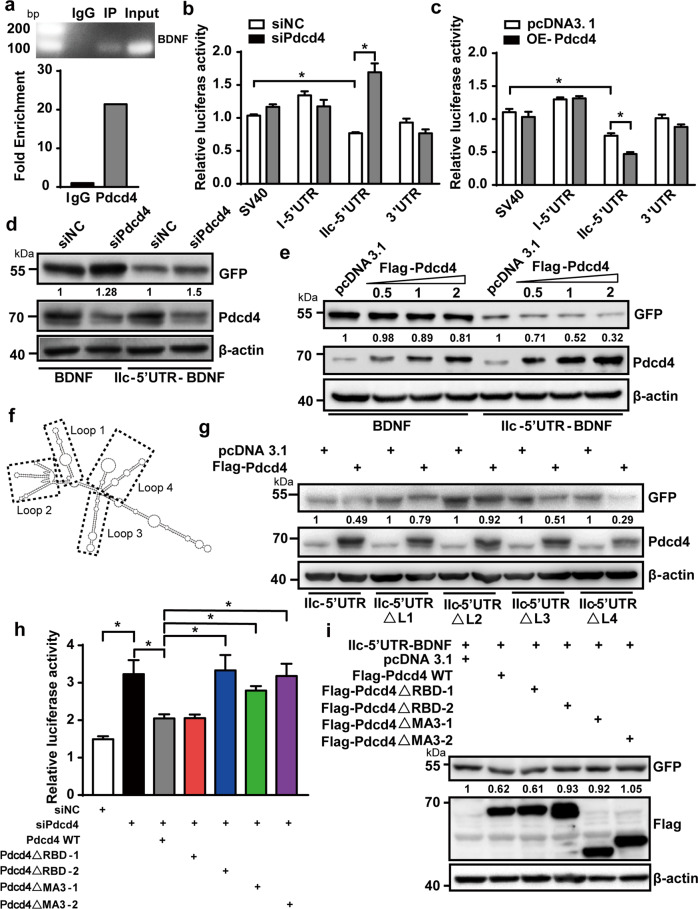
Pdcd4 selectively represses the translation of BDNF splice variant IIc mRNA in an eIF4A-dependent manner. **a** Mice hippocampal lysates were subjected to RNA-IP with Pdcd4 antibodies or IgG. Purified RNA was analyzed by RT- PCR using specific primer for BDNF. **b** Luciferase assays were performed after transfecting each pGL3 construct into HEK 293 cells transfected with either siNC or siPdcd4. Renilla luciferase vector was co-transfected for normalization. Three independent experiments are shown, mean ± SEM, unpaired two-tailed Student’s *t* test, *P < 0.05. **c** Luciferase assays were performed after transfecting each pGL3 construct into HEK 293 cells transfected with either pcDNA3.1 or Flag-Pdcd4. Renilla luciferase vector was co-transfected for normalization. Three independent experiments are shown, mean ± SEM, unpaired two-tailed Student’s *t* test, *P < 0.05. **d** GFP antibody detected BDNF expression after transfecting each BDNF-GFP construct into HEK 293 cells with either siNC or siPdcd4. **e** GFP antibody detected BDNF expression after transfecting different dose Pdcd4 plasmid with each BDNF-GFP construction. **f** Human IIc-isoform BDNF 5’ UTR secondary structure was predicted in the website: http://rna.tbi.univie.ac.at/, and based on principle of the minimum free energy and base pair probabilities from single RNA sequences. **g** Western blot was performed after transfecting each IIc-5’UTR mutation of BDNF-GFP construct into HEK 293 cells transfected with either pcDNA3.1 or Flag-Pdcd4. **h** Luciferase assays were performed after transfecting both pGL3-BDNF variant IIc-5’UTR and siPdcd4 constructions into HEK 293 cells transfected with Pdcd4 mutations. Renilla luciferase vector was co-transfected for normalization. Three independent experiments are shown, mean ± SEM, unpaired two-tailed Student’s *t* test. *P < 0.05. **i** IIc-5’UTR-BDNF-GFP construction with Pdcd4 mutations were transfected into HEK 293 cells, and GFP antibody detected BDNF expression.

To further investigate the mechanisms underlying Pdcd4-mediated BDNF expression, we performed the RNA-immunoprecipitation assay and observed a strong interaction between Pdcd4 and BDNF mRNAs in the hippocampal tissue (Fig. 3a, Supplementary Fig. 6a). As BDNF mRNA has many splice variants which mainly differ from the 5’ UTR, we next investigated whether Pdcd4 inhibited the translation of BDNF splice variant mRNAs. The BDNF mRNA splice variant I and IIc played an important role in the antidepressant effect, and the translation of BDNF mRNA was controlled by its 5’UTR and 3’UTR [32, 33]. Therefore, we separately constructed the 5’ UTR of BDNF variant I and IIc mRNA and the 3’UTR into the luciferase reporter gene plasmid pGL3-enhancer vector (Supplementary Fig. 6b). Each of the luciferase reporter gene plasmid was transfected into the HEK293 cells with either control (siNC) or Pdcd4 siRNA (siPdcd4). We first determined the efficiency of siPdcd4 on the expression of Pdcd4 in HEK293 cells (Supplementary Fig. 6c). Interestingly, the reporter gene harboring the 5’ UTR of BDNF variant IIc mRNA exhibited lower level of luciferase activity than SV40 control group, and which was reversed by Pdcd4 knockdown (Fig. 3b). Conversely, overexpression of Pdcd4 reduced the luciferase activity of reporter gene containing the 5’UTR of the variant BDNF IIc mRNA (Fig. 3c). Collectively, these results suggest that Pdcd4 selectively interacts with the 5’ UTR of the variant BDNF IIc mRNA and suppresses its translation. To further confirm the role of Pdcd4 on BDNF mRNA translation, we constructed the BDNF IIc isoform cDNA containing 5’UTR into the EGFP expression vector and transfected the plasmid into HEK293 cells (Supplementary Fig. 6d). BDNF IIc isoform 5’UTR-carried GFP showed significantly decreased expression, compared with non-5’UTR group. As expected, knockdown of Pdcd4 significantly increased the level of 5’UTR-carried GFP (Fig. 3d). In contrast, overexpression of Pdcd4 decreased the expression of 5’UTR-carried GFP in a dose-dependent manner (Fig. 3e). These data demonstrate that Pdcd4 selectively represses the translation of splice variant BDNF IIc mRNA. We further investigated the effect of Pdcd4 on truncated version of the BDNF IIc form 5’UTR to prove if the ability of Pdcd4 to inhibit translation correlates with the secondary structure of the 5’UTR (Fig. 3f). The results showed that the loop 2 deletion of BDNF IIc mRNA 5’ UTR blocked the inhibitory effect of Pdcd4 on GFP expression (Fig. 3g), which was similar to the 5’UTR of P53 as referred before [22]. The ability of Pdcd4 to suppress BDNF mRNA translation depends on the loop2-like secondary structure-forming potential of the 5’UTR. We also predicted the secondary structure of the 5’UTR in BDNF variant I mRNA and found an obviously different secondary structure with that of IIc mRNA (Supplementary Fig. 7a), further supporting the notion that Pdcd4 affects BDNF mRNA translation via a selective 5’UTR-dependent manner. We next examined whether the inhibitory effect of Pdcd4 on BDNF mRNA translation depends on its interaction with eIF4A. The full-length Pdcd4 contains four significant domains, including two RNA-binding domains (RBD) and two MA3 domains. Pdcd4 mutants were constructed in which four domains were sequentially deleted (Supplementary Fig. 7b). We then co-transfected the mutant or full-length Pdcd4 gene, the siRNA against Pdcd4 and the luciferase reporter gene plasmid containing 5’ UTR of BDNF IIc mRNA into the HEK293 cells. The results showed that knockdown of the endogenous Pdcd4 by siRNA significantly enhanced the luciferase activity, which could be abolished by overexpression of the mouse-derived full-length Pdcd4 (Fig. 3h). It is worth noting that the mutant mouse Pdcd4-∆RBD-2, Pdcd4-∆MA3–1 and the Pdcd4-∆MA3–2 display an increased luciferase activity when compared with the Pdcd4-WT overexpression group (Fig. 3h), indicating that Pdcd4 represses the translation of BDNF IIc mRNA depending on the RBD-2 and the two MA3 domains. Consistently, overexpression of the full length or RBD-1 domain deleted Pdcd4 also significantly decreased the level of BDNF IIc 5’UTR contained GFP (Fig. 3i). It is shown that Pdcd4 inhibits translation by interacting tightly with the RNA helicase eIF4A via its tandem MA3 domains. To study whether Pdcd4 inhibits the translation of BDNF IIc mRNA depending on eIF4A, a plasmid expressing isoform I of eIF4A was constructed, and was co-transfected with plasmids expressing Pdcd4 and BDNF IIc 5’UTR contained GFP into HEK293 cells. It was shown that overexpression of eIF4A-I reversed Pdcd4 induced down-regulation of GFP in a dose-dependent manner (Supplementary Fig. 7c). Moreover, we generated siRNAs to knockdown the expression of the three isoforms eIF4A including I, II and III (Supplementary Fig. 7d). The data showed that reduction of eIF4A by siRNA blocked Pdcd4 knockdown-induced up-regulation of BDNF IIc 5’UTR contained luciferase gene expression (Supplementary Fig. 7d). Above all, these results indicate that Pdcd4 represses IIc isoform BDNF mRNA translation in an eIF4A-dependent manner.

### Pharmacological inhibition of the BDNF signaling abolishes the antidepressant effects in neuron-specific Pdcd4 KO mice in response to CRS

**Fig. 4:**
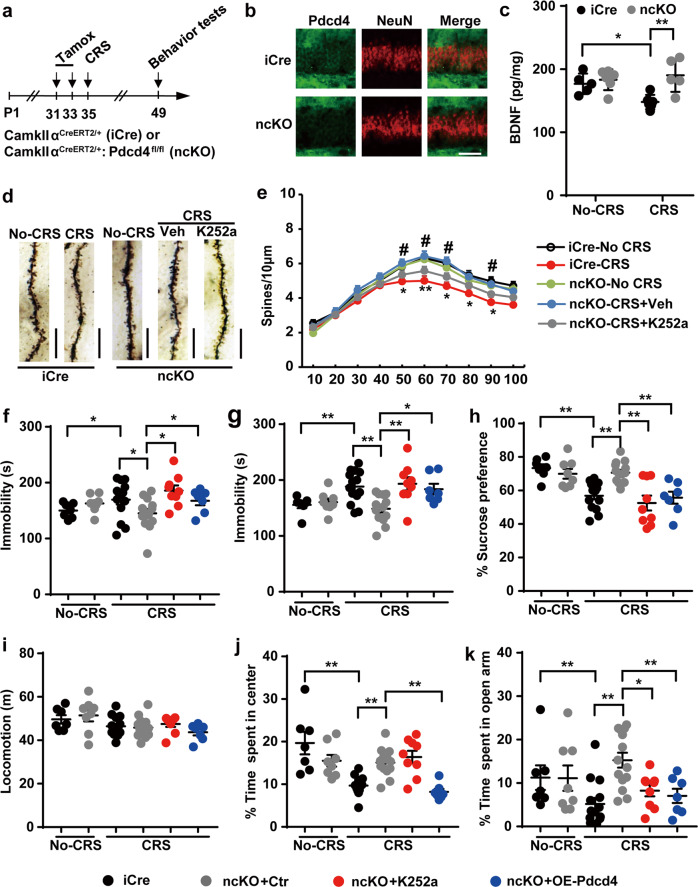
Pharmacological inhibition of the BDNF-TrkB signaling pathway induces depression and anxiety-like behaviors in neuron-specific Pdcd4 knockout mice. **a** Time course of tamoxifen/CRS administration and behavior tests. **b** Coronal sections of dentate gyrus (DG) from control or neuron-depleted mice stained for Pdcd4 after tamoxifen administration in iCre mice or ncKO mice scale bars, 50 µm. **c** Quantitative ELISA analysis of pan-BDNF protein expression in the hippocampus in No-CRS and CRS at iCre mice or ncKO mice. *n* = 5–6 per group; mean ± SEM, two-way ANOVA (iCre vs. ncKO, F_1,18_ = 10.09, *P* < 0.01; No-CRS vs. CRS, F_1,18_ = 2.048, *P* = 0.1696; interaction, F_1,18_ = 5.433, *P* < 0.05) and Sidak’s multiple comparison test, *P < 0.05, **P < 0.01. **d** Representative photomicrographs of dendritic spines from DG neurons, Scale bar, 10 μm. **e** Spine density in dendrites of DG neurons in Pdcd4 cKO and Cre mice. *n* = 4 per group; mean ± SEM, two-way ANOVA, *P < 0.05, **P < 0.01 vs. the No-CRS group; ^#^*P* < 0.05, ^##^*P* < 0.01 vs. the CRS-cKO group. **f** Immobility time in TST, mean ± SEM, two-way ANOVA (iCre vs. ncKO, F_1,45_ = 0.2672, *P* = 0.6078; No-CRS vs. CRS, F_1,45_ = 0.1892, *P* = 0.6656; interaction, F_1,45_ = 8.49, *P* < 0.01) and Sidak’s multiple comparison test, *P < 0.05, **P < 0.01. Under stress condition, one-way ANOVA (F_3,40_ = 3.931, *P* < 0.01) and Tukey’s multiple comparison test, *P < 0.05, **P < 0.01. **g** Immobility time in FST, mean ± SEM, two-way ANOVA (iCre vs. ncKO, F_1,45_ = 2.533, *P* = 0.1185; No-CRS vs. CRS, F_1,45_ = 5.604, *P* < 0.05; interaction, F_1,45_ = 16.13, *P* < 0.01) and Sidak’s multiple comparison test, *P < 0.05, **P < 0.01. Under stress condition, one-way ANOVA (F_3,40_ = 6.553, *P* < 0.01) and Tukey’s multiple comparison test, *P < 0.05, **P < 0.01. **h** Sucrose consumption in SPT. Mean ± SEM, two-way ANOVA (iCre vs. ncKO, F_1,45_ = 5.085, *P* < 0.05; No-CRS vs. CRS, F_1,45_ = 17.32, *P* < 0.01; interaction, F_1,45_ = 20.25, *P* < 0.01) and Sidak’s multiple comparison test, *P < 0.05, **P < 0.01. Under stress condition, one-way ANOVA (F_3,40_ = 9.42, *P* < 0.01) and Tukey’s multiple comparison test, *P < 0.05, **P < 0.01. **i**, **j** Locomotion and time spent in center in the open field test. Mean ± SEM, two-way ANOVA (iCre vs. ncKO, F_1,45_ = 0.6502, *P* = 0.4244; No-CRS vs. CRS, F_1,45_ = 17.32, *P* < 0.01; interaction, F_1,45_ = 14.23, *P* < 0.01) and Sidak’s multiple comparison test, *P < 0.05, **P < 0.01. Under stress condition, one-way ANOVA (F_3,40_ = 17.29, *P* < 0.01) and Tukey’s multiple comparison test, *P < 0.05, **P < 0.01. **k** Time spent in open arm in the elevated plus maze. Mean ± SEM, two-way ANOVA (iCre vs. ncKO, F_1,45_ = 1.914, *P* = 0.1734; No-CRS vs. CRS, F_1,45_ = 2.057, *P* = 0.1584; interaction, F_1,45_ = 11.3, *P* < 0.01) and Sidak’s multiple comparison test, *P < 0.05, **P < 0.01. Under stress condition, one-way ANOVA (F_3,40_ = 9.218, *P* < 0.01) and Tukey’s multiple comparison test, *P < 0.05, **P < 0.01.

To further investigate whether neuron-derived Pdcd4 influences neural function and depression-like behaviors, we generated Pdcd4 conditional knockout mice. We first employed CRISPR/Cas9 to construct Pdcd4^fl/fl^ mice in which the flanked loxP sequences were inserted to exon 4 of the Pdcd4 gene (Supplementary Fig. 8a). To easily examine the knockout efficiency, Pdcd4^fl/fl^ mice were crossed with Eiia-Cre mice which carried Cre-recombinase expression at whole mice body. PCR genotyping results showed that both loxP sequence and Cre gene were existed at mice genome, and further western blot data confirmed the deletion of Pdcd4 protein expression at lung, liver, brain and spleen of Pdcd4^fl/fl^×Eiia-Cre double-positive mice (Supplementary Fig. 8b, c). Next, mice carrying loxP-flanked Pdcd4 were crossed with mice expressing tamoxifen-inducible CamkIIα-driven Cre-recombinase specifically in forehead neurons (CamkIIα^CreERT2^, iCre) to generating neuronal-specific Pdcd4 knockout mice (Pdcd4^fl/fl^, ckiiα^CreERT2/+^, ncKO) (Fig. 4a). The expression of Pdcd4 was completely abolished in hippocampal neurons of the ncKO mice after tamoxifen treatment (Fig. 4b). We first confirmed the BDNF expression of ncKO mice at CRS condition, and found CRS reduced the protein levels of BDNF in iCre mice, but not in the ncKO mice (Fig. 4c). Under non-stressed conditions, immobility and sucrose consumption of ncKO mice (*n* = 7) were similar with those of iCre mice (*n* = 8) (Fig. 4f–h). Meanwhile, ncKO didn’t show anxiolytic behaviors in the OFT and EPM when compared with iCre (Fig. 4i–k). Different from CRS-treated-iCre mice (*n* = 14); ncKO mice (*n* = 14) exposed to CRS showed decreased immobility in TST, FST and increased sucrose consumption in SPT (Fig. 4f–h). Furthermore, after CRS, ncKO mice displayed significant increase of time spent in the center zone in OFT and in the open arms in EPM compared with the iCre group, indicating an anxiolytic effect of ncKO mice (Fig. 4i–k).

To further confirm a direct role of Pdcd4-mediated BDNF-TrkB signaling pathway in CRS-induced emotional disorders, we next investigated whether blockage of BDNF could boost depression in CRS-treated Pdcd4 ncKO mice. To solve this problem, we employed K252a which was recognized TrkB receptor inhibitor. K252a reduced the spine density of hippocampal DG neurons when compared with the vehicle-treated Pdcd4 ncKO mice under stress situation (Fig. 4d, e). Consistent with the morphological results, under CRS condition, repeated infusion of K252a abolished the anti-depressive effect on ncKO mice (*n* = 9), and had blockage of anxiety-like behaviors of ncKO mice (Fig. 4f–k). To pin down whether the behavioral changes of ncKO mice were caused by hippocampus-expressed Pdcd4, we injected AAV9 virus-containing Pdcd4 into the ventral hippocampus. The behavioral test results showed that overexpression of Pdcd4 into ncKO mice (*n* = 7) attenuated the CRS resilience character of ncKO mice (Fig. 4f–k). Taken together, these results indicate that the major depression-regulation role of Pdcd4 in hippocampal neuron relies on the BDNF-TrkB signaling.

### Knockdown of Pdcd4 in hippocampus suppresses CRS-induced depression-like behaviors

To address the clinical significance of Pdcd4 in major mood disorders in human, we screened the gene expression profiles in the postmortem brain of patients with bipolar disorder, major depression and schizophrenia from the GEO database (GSE42546, GSE53987 and GSE12654), and then analyzed the relation between Pdcd4 expression level and diseases (Supplementary Table 4). Collectively, we found that the levels of Pdcd4 increased in patients with schizophrenia and MDD compared with control subjects in hippocampus (Supplementary Fig. 9), suggesting that the excessive expression of Pdcd4 might be an important factor that contributes to the development of human MDD. To address the potential value of Pdcd4 as a target of anti-depressive therapy, we investigated whether knockdown of Pdcd4 with siRNA could prevent or rescue CRS-induced depression- and anxiety-like behaviors in mice. For prevention, lentivirus containing Pdcd4 siRNA (siPdcd4) and GFP was injected into the hippocampus of mice before CRS (Virus + CRS) and for the rescue experiment, lentivirus expressing siPdcd4 and GFP was administrated after the CRS treatment (CRS + Virus) (Supplementary Fig. 10a). The control mice received microinjection of lentivirus only expressing GFP (siNC). Lentivirus containing siPdcd4 effectively reduced the expression of Pdcd4 in the ventral hippocampus (Supplementary Fig. 10b, c). Next, the diffusion of virus was detected by immunofluorescent staining of GFP at day 14 (Supplementary Fig. 10d). Notably, reduction of Pdcd4 significantly attenuated the CRS-increased immobility of mice in the TST and FST experiments, no matter the virus was injected before or after CRS (Supplementary Fig. 10e, f). Moreover, in the SPT, CRS exposure caused less sucrose consumption in mice injected with control virus, but had no effect on mice injected with virus-containing siPdcd4 (Supplementary Fig. 10g). These data suggest that knockdown of Pdcd4 in the hippocampus prevents and rescues CRS-induced depression-like behaviors in mice. We next examined the effect of Pdcd4 knockdown on CRS-induced anxiety-like behaviors. CRS-induced significant reduction of time spent in the center in the OFT and time spent in the open arms in the EPM in the siNC virus injected control mice (Supplementary Fig. 10h–j). However, when the virus-containing siPdcd4 was injected before CRS, the mice would spend significantly more time in the center in the OFT and in the open arms in the EPM compared with those injected with control virus, suggesting that knockdown of Pdcd4 prevented CRS-induced anxiety-like behavior in mice (Supplementary Fig. 10h–j). Unexpected, knockdown of Pdcd4 after CRS could not rescue the anxiety-like behavior caused by CRS in mice, that is different from the effect of Pdcd4 knockdown on depression-like behavior.

### Blockage of the interaction between Pdcd4 and eIF4A prevents CRS-induced depression-like behaviors

**Fig. 5:**
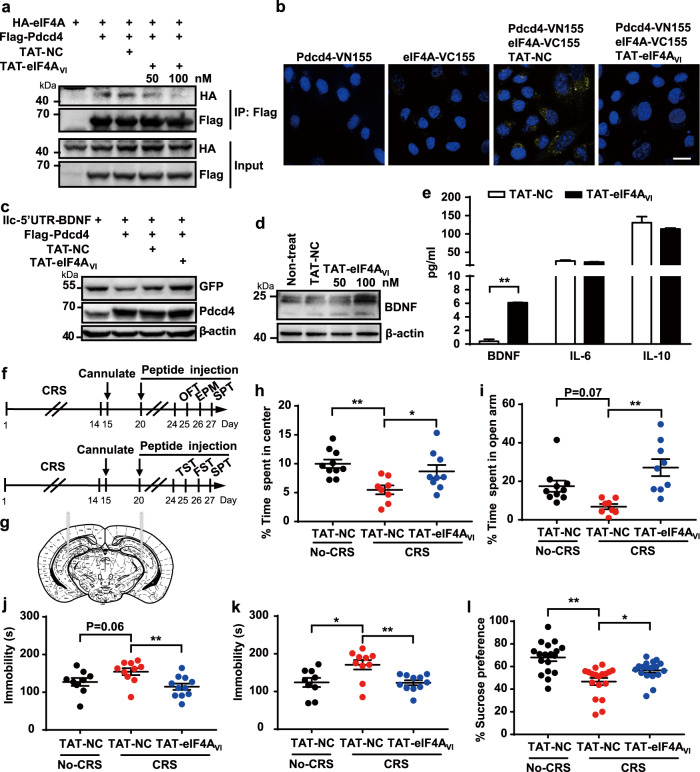
Pdcd4-targeting peptide treatment promotes BDNF expression and has antidepressant response. **a** HEK293 cells were co-transfected with Flag-Pdcd4, HA-eIF4A and peptide. Immunoprecipitation was performed with the anti-Flag antibody. Immunoblotting was performed with anti-Flag or anti-HA antibodies. The figure represents three independent experiments that yield similar result. **b** HEK293 cells were transfected with BiFC plasmids. Pictures showed DAPI staining (Blue) and Venus (Yellow). Scale bar, 10 μm. **c** GFP antibody detected BDNF expression after transfecting each IIc-5’UTR-BDNF-GFP construct into HEK 293 cells with either TAT-NC or TAT-eIF4A_VI_. **d** The level of BDNF expression in neurons after TAT-NC or TAT-eIF4A_VI_ administration 24 h. **e** ELISA detected the expression of BDNF, IL-10 and IL-6 in neurons’ medium (*n* = 3; **P < 0.01 compared with the TAT-eIF4A_VI_ group). **f** Time course of peptide injection/CRS administration and behavior tests. **g** Representative photomicrographs of injection sites in the hippocampus. **h** Time spent in center in the open field test. *n* = 8–10 per group; mean ± SEM, unpaired two-tailed Student’s *t* test. **i** Time spent in open arm in the elevated plus maze. *n* = 8–10 per group; mean ± SEM, unpaired two-tailed Student’s *t* test. **j** Immobility time in TST, **k** Immobility time in FST. *n* = 9–11 per group; mean ± SEM, unpaired two-tailed Student’s *t* test, *P < 0.05 and **P < 0.01. **l** Sucrose consumption in SPT. *n* = 18 per group; mean ± SEM, unpaired two-tailed Student’s *t* test, *P < 0.05 and **P < 0.01.

As Pdcd4 suppresses the expression of BDNF in an eIF4A-dependent manner (Supplementary Fig. 7), we speculated that if interrupting the Pdcd4-eIF4A interaction could exert the BDNF expression and produce antidepressant-like effects. To develop a peptide that disrupts the two proteins interaction, we selected four candidate motif of eIF4A, which were found to substantial Pdcd4 interaction regions [34], and identified VI motif (His358-Arg365) is necessary for the complex formation. We thus generated the peptide of TAT-eIF4A_VI_ and transfected into HEK293 cells. The data showed that TAT-eIF4A_VI_ obviously disrupted the interaction between Pdcd4 and eIF4A (Fig. 5a). Further, BiFC data analysis showed that Venus fluorescence was significantly diminished after TAT-eIF4A_VI_ administration (Fig. 5b). Importantly, we found that TAT-eIF4A_VI_ promoted BDNF expression in a dose depend manner (Fig. 5c, d). To confirm this result, we tested the effect of TAT-eIF4A_VI_ in cultured neurons, and found that the TAT-eIF4A_VI_ robustly increased the expression of BDNF but not of IL-6 and IL-10 (Fig. 5e). Taken together, these data demonstrate that targeting on Pdcd4-eIF4A association with specific peptide promotes BDNF expression at post-transcriptional level. To verify the antidepressant effect of TAT-eIF4A_VI_ in CRS model, TAT-NC or TAT-eIF4A_VI_ was injected into the hippocampus of WT mice (Fig. 5f, g). TAT-NC injected mice displayed obviously increased anxiety-like behavior after CRS as shown by decreased time spent in the center in OFT and in the open arm in EPM while the TAT-eIF4A_VI_ injected mice showed no obvious alterations in response to CRS (Fig. 5h, i). As the TST, FST, SPT results shown, we found that the administration of TAT-eIF4A_VI_ significantly attenuated the immobility and increased the sucrose preference of mice after CRS (Fig. 5j–l). Overall, these data indicate that blockage of the association between Pdcd4 and eIF4A by TAT-eIF4A_VI_ prevents mice to suffer from CRS-induced anxiety- and depression-like behaviors.

## Discussion

The emergence of the antidepressant effect of promoting BDNF translation had been previously noticed. To clarify the regulatory mechanism of BDNF expression is vital for exploring the new methods for antidepressant therapy. Numerous researches focus on genetic and epigenetic modulation of BDNF expression in MDD [35]. However, the molecular mechanism of BDNF protein expression in depression is still obscure. In this article, we provide direct evidence that Pdcd4 impairs synaptic plasticity and consequently results in the depression-like behaviors through suppression of BDNF mRNA translation in response to CRS, suggesting Pdcd4 might be a potential target for depression therapy.

Our work first reveals that Pdcd4 selectively repressed the translation of variant IIc BDNF mRNA, which is involved in depression. CRS decreased the expression of BDNF mRNA and protein in WT mice. However, Pdcd4 KO mice were resilient to CRS induced the reduction of protein level of BDNF. Based on this observation, we discover that Pdcd4 inhibit BDNF mRNA translation during CRS. Pdcd4 is a translational repressor, and it blocks the translation of target mRNAs depending on their 5’UTR structure [18]. It is reported that there are several isoforms of BDNF mRNA and the translation of BDNF mRNA depends on the variant 5’ UTR and the 3’UTR regions [32]. The BDNF 3’UTR have two structures, short-form and long-form. The two forms of 3’UTR, respectively, control BDNF subcellular distribution and regulate mRNA local translation with the facility of HuD and BicD2 [35–37]. Previously, it has been reported that BDNF 5’UTR has the distinct tissue-specific expression profiles in both mouse and rat [38]. The BDNF IIc variant mRNA is widely expressed in cortex and hippocampus, exhibiting sensitive to KCl and antidepressant treatment [33]. However, the function of 5’UTR of BDNF mRNA on its translation is still unknown. It has been proved the motif of 5’UTR that contains several RNA nucleotides contributes to mRNA translation. We predicted the structure of 5’UTR of BDNF I and IIc mRNA and found that the secondary structure of IIc type mRNA was a target mRNA of Pdcd4. Thus it concludes that Pdcd4 regulates mRNA translation in a secondary structure of 5’UTR-dependent manner. In addition, the inhibitory effect of Pdcd4 on BDNF mRNA translation depends on eIF4A, which extends our understanding of the mechanisms underlying the vital role of mRNA translation in depression regulation. Based on that translational mechanism, we devote to find a new antidepressant treatment by controlling BDNF in depression. As observed by previous studies, the antidepressant effect of ketamine and other NMDA receptor antagonist, MK801, required the expression of BDNF and trigger BDNF translational machinery [17]. Our results support the idea of the mechanism of the antidepressant effect, and we discover that there is a direct way for manipulating BDNF expression at post-transcriptional level in depression. We found that Pdcd4 meditated the function of mTORC1-BDNF axis in depression. The activation of the mTORC1 signaling pathway is necessary for antidepressant responses, and drugs that directly target the mTORC1 pathway should be an option of interest for MDD treatment. However, possible side effects from persistent trigging of the mTORC1 activity increase the risk for uncontrolled cell proliferation and tumors [39]. Therefore, we develop a new peptide, TAT-eIF4A_VI_, specifically targeting the Pdcd4-eIF4A complex which is the key node of BDNF translation. By blocking Pdcd4-eIF4A interaction, BDNF protein level is up-regulated, opening the possibility of developing a therapy, particularly for those diseases in which the pathogenesis is caused by loss of BDNF.

Second, our data provide a novel insight about Pdcd4 in the development of depression. Pdcd4 plays a critical role in the pathogenesis of depressive disorder, and thus is a potential target of antidepressant. Supporting this conclusion included that Pdcd4 was predominantly expressed by neuron rather than astrocytes in mouse brain, and the level of Pdcd4 expression was increased in the hippocampus after CRS. Consistently, NCBI GEO database profiles showed that patients with depression had higher Pdcd4 expression in the hippocampus. Meanwhile, Pdcd4 KO mice had no changes in gross neuroanatomical and morphological structure of brain. Interestingly, neuron-specific Pdcd4 KO mice showed similar behavioral alternations as Pdcd4 global knockout mice. Though the function of Pdcd4 in neurons needs further study, some evidence pointed out that Pdcd4 is a stress-sensitive molecule in neurons. It has been found that EtOH or UV exposure caused an increased expression of Pdcd4 in neurons, and that up-regulation contributed to stress granules formation [40, 41]. CRS is a well-established animal model to induce depression and anxiety-like behaviors in mice, which mimic the environment overburden [42]. Consistent with these findings, we find that manipulation of Pdcd4 expression in neuron is contributed to depression regulation. Currently, fluoxetine, sertraline and imipramine are used for depression treatment due to their selective serotonin reuptake inhibition character. However, many patients with depression show no response or delayed effects to the antidepressant therapies [43]. Thus, manipulating the alterations of genes associated with depression is perspective for the treatment depressive disorder. Pdcd4 is a potential target of antidepressant therapy, for the low side-effect due to its non-effect at baseline.

Finally, we find that Pdcd4 mediates mTORC1-related synaptic plasticity. Synaptic plasticity, the activity-dependent modulation of the strength of synaptic communications, underlies changes in neuronal network dynamics and is therefore thought to be involved in processing emotional message [44]. New synaptic connection formation requires new protein synthesis. For the function of translation regulation, mTORC1 signaling pathway plays an essential role in neuronal excitability, neuronal survival, synaptic plasticity, cognition and emotional behavior in brain [39, 45]. Chronic unpredictable mild stress (CUMS) induced the mTORC1 signaling reduction was attributed to increased expression of REDD1, a negative regulator of mTORC1, increasing expression, and the changes of that was also confirmed in the PFC of human subjects with depression [30]. Moreover, the mTORC1 signaling inactivation led to dendritic re-organization and loss of spines in the PFC as well as the hippocampus, which was supported by a report that spine atrophy in postmortem PFC of depressive subjects resulted from the decreased level of S6K1 [46]. Conversely, ketamine, a kind of fast-acting antidepressant, rapidly reversed these synaptic deficits of depression via trigging mTORC1/S6K1 signaling [47]. Therefore, mTORC1 signaling is an essential signaling pathway in synaptic plasticity regulation in depression. Herein, we uncovered the involvement of Pdcd4, a mTORC1 downstream effector, in depression regulation. We found that Pdcd4 was phosphorylated at serine 67 site by S6K1 upon mTORC1 signaling activation, and the phosphorylation contributed to Pdcd4 overexpression at stress condition [24], suggesting that mTORC1 plays a key role in emotional regulation due to Pdcd4’s attribution. In addition, we discovered an increased phosphorylation expression of S6 in the hippocampus of Pdcd4 KO mice, and rapamycin partially inhibited Pdcd4 KO-induced S6 over-activation (Supplementary Fig. 5b). It indicates that there is a Pdcd4 negative-regulation mechanism involving S6K/S6 signaling. In accordance with previous research, the association of Pdcd4-Rictor complex, an exclusive component of mTORC2, contributed to Akt-IKK-mTORC1 axis and then blocked mTORC1 signaling activation [48]. Therefore, it is probably that mTORC2 complex also plays a part in Pdcd4-regulated depression-like behaviors. Less evidence has referred the function of mTORC2 in depression; except one recent research pointed that mTORC2 is required for hippocampal mGluR-LTD and related memory behavior [49]. Future studies should focus on the effect of mTORC2 in depression.

In summary, the present study identifies a critical role in mTORC1-Pdcd4 axis mediating BDNF mRNA translation in the hippocampus for the impairment of neuronal plasticity and depression-like behaviors in mice in response to chronic stress (Supplementary Fig. 11). Our findings have provided a novel insight into the role of Pdcd4 in stress-induced emotional disorders, suggesting Pdcd4 might be an effective option for the treatment of depressive disorder.

## Supplementary information

Supplementary Table 1

Supplementary Table 2

Supplementary Table 3

Supplementary Table 4

Supplementary Figures
