# Supplementary material for: Programmed cell death 4 as an endogenous suppressor of BDNF translation is involved in stress-induced depression
Source: Mol Psychiatry. 2020 Mar 16;26(6):2316–33. doi: 10.1038/s41380-020-0692-x (PMC8440200; doi:10.1038/s41380-020-0692-x)
Supplement: Supplementary file 1 — Supplementary Table 1 [file 41380_2020_692_MOESM1_ESM.docx]

**Supplementary Table1: Antibodies used in this study.**

| **Primary antibodies** | **Host** | **Dilution and supplier** | **Company and catalog** |
| --- | --- | --- | --- |
| Pdcd4 | Rabbit | 1:2000 WB  1:100 IHC  1:200 IP | Cell Signaling Technology, Beverly, MA, USA; Cat: 9535 |
| p-Pdcd4 S67 | Rabbit | 1:1000 WB | Abcam, Cambridge, MA,USA; Cat: ab73343 |
| β-actin | Mouse | 1:1000 WB | Sigma-Aldrich, St.Louis. MO,USA; Cat: A5441 |
| p-mTOR | Rabbit | 1:1000 WB | Cell Signaling Technology, Beverly, MA, USA; Cat: 5536 |
| mTOR | Rabbit | 1:2000 WB | Cell Signaling Technology, Beverly, MA, USA; Cat: 2983 |
| p-S6 | Rabbit | 1:1000 WB | Cell Signaling Technology, Beverly, MA, USA; Cat: 5364 |
| S6 | Rabbit | 1:1000 WB | Cell Signaling Technology, Beverly, MA, USA; Cat: 2217 |
| GFP | Rabbit | 1:2000 WB | Invitrogen, Carlsbad, CA,USA; Cat: A11122 |
| Ub | Mouse | 1:1000 WB | Santa Cruze, Dallas, Texas,  USA; Cat: sc-8017 |
| Flag | Rabbit | 1:2000 WB | Thermo Fisher, MA,USA; Cat: PA1-984B |
| HA | Rabbit | 1:2000 WB | Sigma-Aldrich, St.Louis. MO,USA; Cat: SAB4301135 |
| GFAP | Mouse | 1:500 IHC | Cell Signaling Technology, Beverly, MA, USA; Cat: 3670 |
| NeuN | Mouse | 1:500 IHC | Millipore, Burlington, MA, USA; |
| eIF4A | Rabbit | 1:1000 WB | Cell Signaling Technology, Beverly, MA, USA; Cat: 2490 |
