# Supplementary material for: Programmed cell death 4 as an endogenous suppressor of BDNF translation is involved in stress-induced depression
Source: Mol Psychiatry. 2020 Mar 16;26(6):2316–33. doi: 10.1038/s41380-020-0692-x (PMC8440200; doi:10.1038/s41380-020-0692-x)
Supplement: Supplementary file 3 — Supplementary Table 3 [file 41380_2020_692_MOESM3_ESM.docx]

**Supplementary Table 3: The clinical parameters of postmortem brains–derived individuals used in this study**

| **GEO code** | **Subject** | **Age (years)** | **Gender**  **(Male :**  **female)** | **PMI (h)** | **RIN** | **Cause of death**  **(Suicide :**  **Nonsuicide)** |
| --- | --- | --- | --- | --- | --- | --- |
| GSE42546 | Control | 48.1 (29-68) | 9M : 6F | 23.7 (8-42) | - | 0S : 15NS |
|  | Bipolar disorder | 42.3 (25-61) | 9M : 6F | 32.5 (13-62) | - | 9S : 6NS |
|  | Major depression | 46.4 (30-65) | 9M : 6F | 27.5 (7-47) | - | 7S : 8NS |
|  | Schizophrenia | 44.2 (25-62) | 9M : 6F | 33.7 (12-61) | - | 4S : 11NS |
| GSE53987 | Control | 48.1+10.6 | 10M : 9F | 19.5+5.1 | PFC 7.8+ 0.6  HIP 6.4+ 0.5 | 0S : 19NS |
|  | Bipolar disorder | 46.3+9.5 | 10M : 9F | 21.3+6.6 | PFC 7.6+ 0.6  HIP 6.4+ 0.7 | 8S : 11NS |
|  | Major depression | 45.2+10.1 | 10M : 9F | 20.1+6.0 | PFC 7.7+ 0.5  HIP 6.3+ 0.4 | 7S : 12NS |
|  | Schizophrenia | 45.1+8.5 | 10M : 9F | 20.1+6.9 | PFC 7.7+ 0.7  HIP 6.0+ 0.6 | 7S : 12NS |
| GSE12654 | Control | 48+11 | 9M :6F | 24+10 | - | 0S : 15NS |
|  | Bipolar disorder | 39+12 | 8M : 3F | 32+16 | - | 9S : 2NS |
|  | Major depression | 46+10 | 6M : 5F | 27+12 | - | 9S : 2NS |
|  | Schizophrenia | 44+14 | 8M : 5F | 33+15 | - | 10S : 3NS |
