# Supplementary material for: Programmed cell death 4 as an endogenous suppressor of BDNF translation is involved in stress-induced depression
Source: Mol Psychiatry. 2020 Mar 16;26(6):2316–33. doi: 10.1038/s41380-020-0692-x (PMC8440200; doi:10.1038/s41380-020-0692-x)
Supplement: Supplementary file 4 — Supplementary Table 4 [file 41380_2020_692_MOESM4_ESM.docx]

| **Region** | **GEO code** | **Subject** | **Mean**  **±SEM** | **Mean of Fold change**  **±SEM** | **P value** |
| --- | --- | --- | --- | --- | --- |
| Hippocampus | GES42546 | Control | 63.37±3.52 | 1.00±0.06 | - |
|  |  | Bipolar disorder | 71.82±4.85 | 1.13±0.08 | 0.16 |
|  |  | Major depression | 74.11±1.99 | 1.17±0.03 | 0.03* |
|  |  | Schizophrenia | 75.74±2.91 | 1.20±0.05 | 0.02* |
|  | GES53987 | Control | 51.02±1.60 | 1.00±0.03 | - |
|  |  | Bipolar disorder | 53.98±2.50 | 1.06±0.05 | 0.32 |
|  |  | Major depression | 54.37±1.83 | 1.07±0.04 | 0.18 |
|  |  | Schizophrenia | 56.24±2.68 | 1.10±0.05 | 0.09 |
|  | Total | Control | - | 1.00±0.04 | - |
|  |  | Bipolar disorder | - | 1.09±0.04 | 0.10 |
|  |  | Major depression | - | 1.11±0.03 | 0.016* |
|  |  | Schizophrenia | - | 1.15±0.04 | 0.005** |
| Prefrontal Cortex | GES12654 | Control | 0.18±0.04 | 1.00±0.22 | - |
|  |  | Bipolar disorder | 0.23±0.03 | 1.30±0.21 | 0.20 |
|  |  | Major depression | 0.21±0.03 | 1.18±0.21 | 0.45 |
|  |  | Schizophrenia | 0.15±0.02 | 0.83±0.15 | 0.48 |
|  | GES53987 | Control | 21.09±0.82 | 1.00±0.04 | - |
|  |  | Bipolar disorder | 23.44±1.65 | 1.11±0.04 | 0.20 |
|  |  | Major depression | 23.02±1.39 | 1.09±0.04 | 0.22 |
|  |  | Schizophrenia | 20.00±1.80 | 0.95±0.09 | 0.54 |
|  | Total | Control | - | 1.00±0.09 | - |
|  |  | Bipolar disorder | - | 1.20±0.10 | 0.15 |
|  |  | Major depression | - | 1.12±0.09 | 0.37 |
|  |  | Schizophrenia | - | 0.85±0.08 | 0.25 |

**Supplementary Table 4: Summarized the statistical analysis of the Pdcd4 expression level in human brain**
