# Supplementary material for: Programmed cell death 4 as an endogenous suppressor of BDNF translation is involved in stress-induced depression
Source: Mol Psychiatry. 2020 Mar 16;26(6):2316–33. doi: 10.1038/s41380-020-0692-x (PMC8440200; doi:10.1038/s41380-020-0692-x)
Supplement: Supplementary file 5 — Supplementary Figures [file 41380_2020_692_MOESM5_ESM.docx]

**Supplementary Figure 1. Pdcd4 expression pattern in mice brain.** (a) Mice prefrontal cortex, Hippocampus, Hypothalam, Striaum, Entorhinal cortex, Thalam and Cerebellum tissue were grinded, and western blot detected the level of Pdcd4 protein. Three independent experiences are shown. (b) Immunoﬂuorescence images showing endogenous Pdcd4 expression in the hippocampus. Tissue from Naïve mice were co-immunostained between Pdcd4 (green) and NeuN (red). Scale bars, 50µm. Three independent experiments are shown. (c) Tissues were co-immunostaining between Pdcd4 (green) and GFAP (red). Scale bars, 50µm. Three independent experiments are shown. (d-e) Quantification of Pdcd4 expression level in hippocampal subregions. n = 4 mice per group, **P<0.01 (unpaired two-tailed student’s t test). Values are mean± SEM. The tissue from No-CRS and CRS mice were stained with the Pdcd4 (green). Scale bars, 200µm.

**Supplementary Figure 2. Histological appearance of the Pdcd4 KO mice.** (a-e) Nissle staining in the Pdcd4 KO and WT mice: slices of the prefrontal cortex (a-b) and hippocampus (c-e) collected from the Pdcd4 KO mice showed no gross (a, c) or layered structural (b, d) abnormalities. A higher-magnitude image of the hippocampal CA1, CA3, and DG regions (e). Scale bar, 50μm.

**Supplementary Figure 3.** **Anxious behavior alternation in Pdcd4 KO mice and Pdcd4 overexpressed mice.** (a-b) Locomotion and time spent in center in the open field test. n =7-8 per group; mean± SEM, two-way ANOVA, **P<0.01. (c) Time spent in open arm in the elevated plus maze. n=7-8 per group; mean± SEM, two-way ANOVA, **P<0.01. (d) Mean single subject moving distance (± SEM) in the open field test for control (n=8) and OE-Pdcd4 (n=9) animals (unpaired two-tailed Student’s t test). (e) Mean center zone duration (± SEM) in the open field test for control (n=8) and OE-Pdcd4 (n=9) animals, *P<0.05 (unpaired two-tailed Student’s t test). (f) Mean open-arm duration (± SEM) in the elevated plus maze for control (n=8) and OE-Pdcd4 (n=9) animals, *P<0.05 (unpaired two-tailed Student’s t test).

**Supplementay Figure 4. Alteration of mTORC1 signaling in acute and chronic stress.** (a) Phosphoprotein levels in hippocampus in No-CRS (n=5-6), CRS (n=6-8) mice. (b) Pdcd4 protein ubiquitination in hippocampus in No-CRS (n=4), CRS (n=4) mice. (c) Phosphoprotein levels in hippocampus in No-ARS (n=8), ARS (n=8) mice. Phosphoprotein levels were normalized to total protein levels. (d) Pdcd4 protein expression level in hippocampus No-ARS (n=7), ARS (n=7) mice. Results are the mean± SEM., unpaired two-tailed Student’s t test.

**Supplementay Figure 5. mTORC1 signaling alteration in Pdcd4 KO mice.** (a) Pdcd4 KO and WT mice were injected with Rapamycin during CRS. Hippocampal tissue lysis were applied to detect protein phosphorylation in different groups, n=6 per group. (b) Phosphoprotein levels in hippocampus. n=6 per group.

**Supplementary Figure 6. Plasmid construction.** (a) RNA-IP with anti-sNRNP70 or IgG (negative control) acts as positive control. Purified RNA was measured by RT-PCR using specific primer for positive control U1 snRNA. Three independent experiences are shown. (b) A schematic of luciferase reporter (pGL3) constructs in which the 5’-UTR (pGL3-BDNF variant I-5’UTR and pGL3-BDNF variant IIc-5’UTR), 3’UTR (pGL3-BDNF-3’UTR) were cloned upstream or downstream of the luciferase coding region (Luc). (c) Determining the human siPdcd4 efficiency, siNC or siPdcd4 was transfected into HEK 293 cells. Three independent experiences are shown. (d) A schematic of the constructs in which the mouse BDNF CDS sequence (BDNF-GFP) or IIc variant 5’UTR (IIc 5’UTR-BDNF-GFP) were cloned upstream of the GFP coding region (Luc).

**Supplementary Figure 7. Pdcd4 controlled BDNF translation through eIF4A.** (a) Human BDNF 5’ UTR secondary structure was predicted in the website: <http://rna.tbi.univie.ac.at/>, and based on principle of the minimum free energy and base pair probabilities from single RNA sequences. (b) A schematic of Pdcd4 truncated constructs. (c) GFP antibody detected BDNF expression after transfecting different dose eIF4A plasmid with each BDNF-GFP construction. Three independent experiences are shown. (d) RT-PCR detected eIF4A expression after transfecting each siRNA in HEK 293 cells. Three independent experiences are shown, mean± SEM, one-way ANOVA, *P<0.05. (e) Luciferase assays were performed after transfecting both pGL3-BDNF variant IIc-5’-UTR and siPdcd4 constructs into HEK 293 cells transfected with sieIF4A. Renilla luciferase vector was co-transfected for normalization. Three independent experiences are shown, mean± SEM, one-way ANOVA, *P<0.05, **P<0.01.

**Supplementary Figure 8. The construction of Pdcd4 conditional knockout mice system.** (a) Schematic of the targeting strategy used for knockout of Pdcd4 at the exon 4 locus, and the primers designed for genotyping. (b) PCR genotyping for Pdcd4 conditional knockout mice. (c) By using Pdcd4 specific antibody to detected knockout efficiency at brain, lung, liver, and spleen.

**Supplementary Figure 9. Microarray data analysis from GEO bank.** (a) Human hippocampus relative Pdcd4 expression fold change in different kind of patients from GEO (GSE42546). Ctr (control) n=15, MDD (major depressive disorder) n=15, BD (bipolar disorder) n=15, Sch (schizophrenia) n=15. (b) Human hippocampus relative Pdcd4 expression fold change in different kind of patients from GEO (GSE53987). Ctr (control) n=19, MDD (major depressive disorder) n=19, BD (bipolar disorder) n=19, Sch (schizophrenia) n=19. (c) Human hippocampus relative Pdcd4 expression fold change. Ctr (control) n=34, MDD (major depressive disorder) n=34, BD (bipolar disorder) n=34, Sch (schizophrenia) n=34. (d) Human prefrontal cortex relative Pdcd4 expression fold change in different kind of patients from GEO (GSE12654). Ctr (control) n=15, MDD (major depressive disorder) n=11, BD (bipolar disorder) n=11, Sch (schizophrenia) n=13. (e) Human prefrontal cortex relative Pdcd4 expression fold change in different kind of patients from GEO (GSE53987). Ctr (control) n=19, MDD (major depressive disorder) n=19, BD (bipolar disorder) n=19, Sch (schizophrenia) n=19. (f) Human prefrontal cortex relative Pdcd4 expression fold change. Ctr (control) n=34, MDD (major depressive disorder) n=30, BD (bipolar disorder) n=30, Sch (schizophrenia) n=32. mean± SEM, unpaired two-tailed Student’s t test, *P < 0.05, **P<0.01.

**Supplementary Figure 10. Knockdown of Pdcd4 in hippocampus suppressed CRS-induced depression-like behaviors.** (a) The graph displays lentivirus injection before or after CRS. (b-c) Mean (± SEM) fold changes of Pdcd4 from virus-infused microdissections from siNC (n=4) or siPdcd4 (n=4) mice. unpaired two-tailed Student’s t test, *P<0.05. (d) Representative photomicrographs of injection sites in the hippocampus. Error bar 50 μm (e) Immobility time in TST, (f) immobility time in FST and (g) sucrose consumption in SPT. n=8-16 per group; mean± SEM, two-way ANOVA, *P<0.05 and **P<0.01. (h-i) Locomotion and time spent in center in the open field test. n=8-16 per group; mean± SEM, two-way ANOVA, *P<0.05 and **P<0.01. (j) Mean open-arm duration in the elevated plus maze. n=8-16 per group; mean± SEM, two-way ANOVA, *P<0.05 and **P <0.01.

**Supplementary Figure 11. Working model.** CRS suppressed the activation of mTORC1 signaling and ubiquitin-mediated degradation of Pdcd4, resulting in decreased protein level of BDNF and neuronal spine density in the hippocampus.
